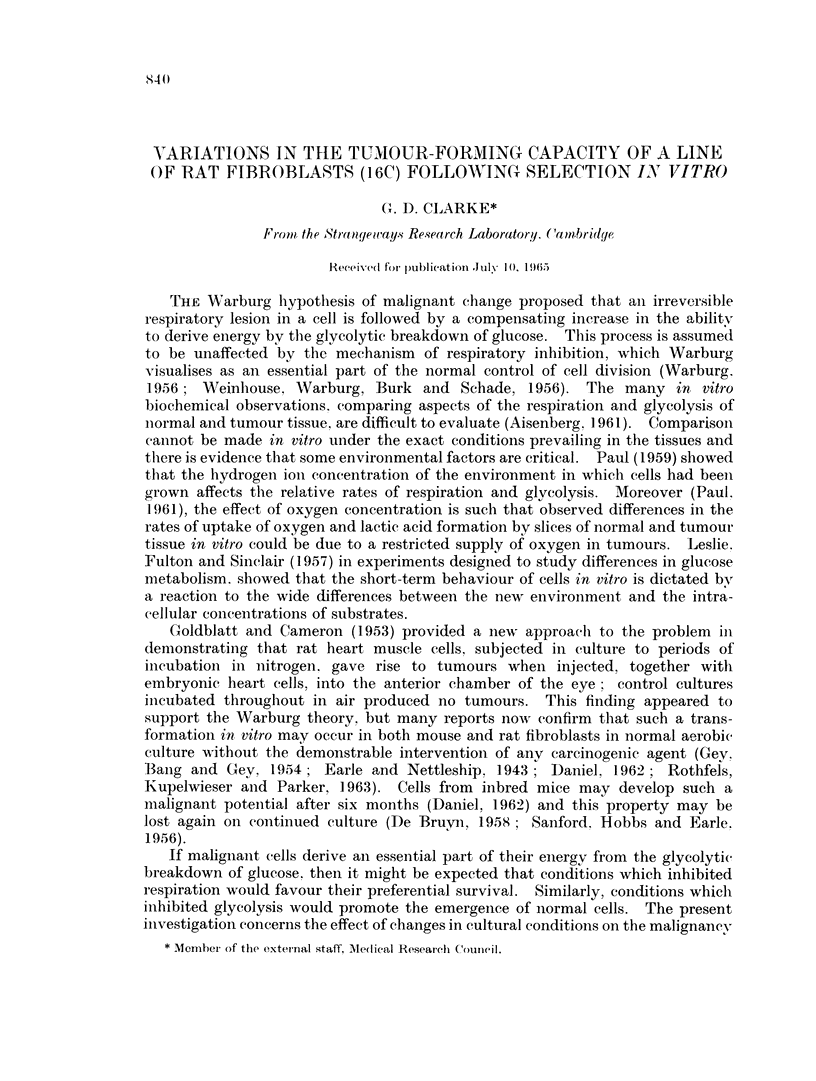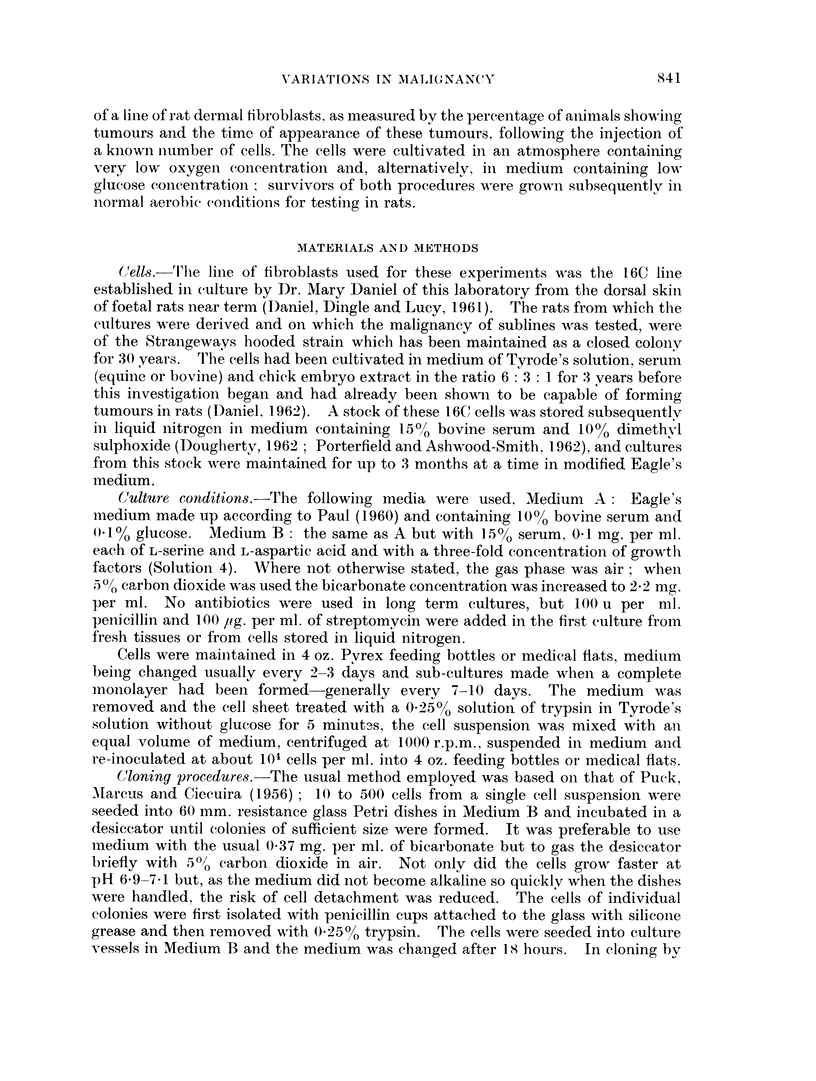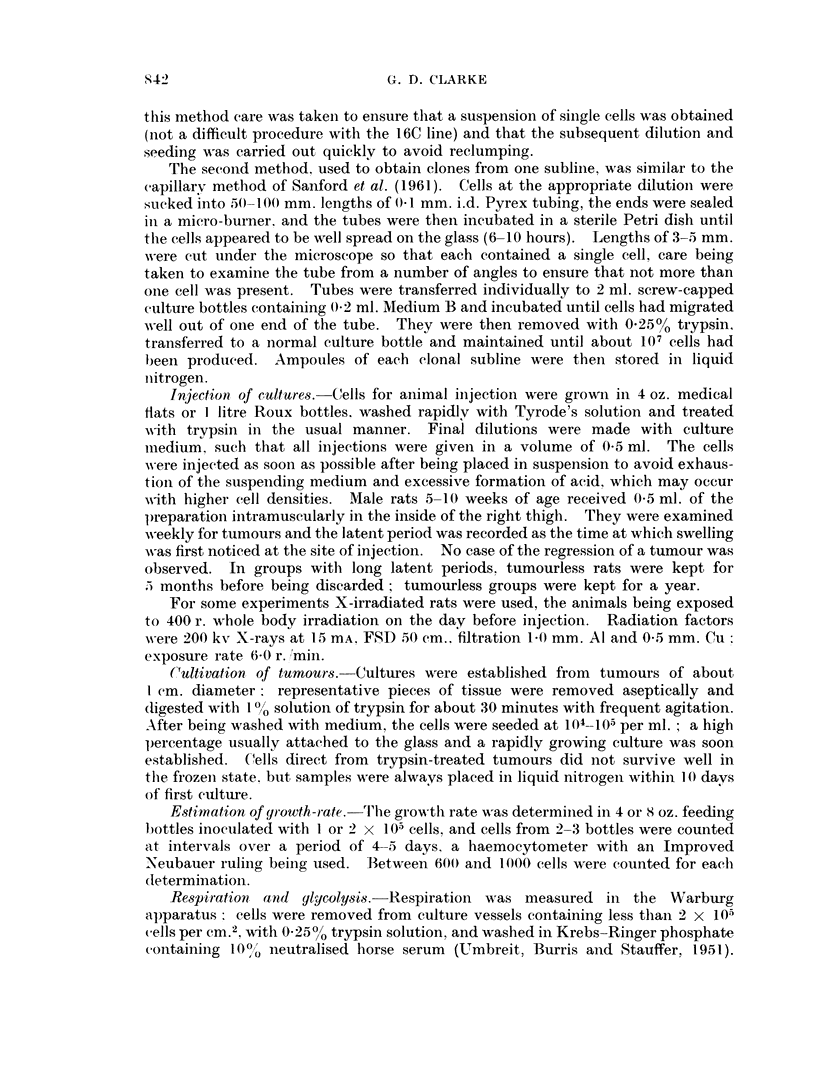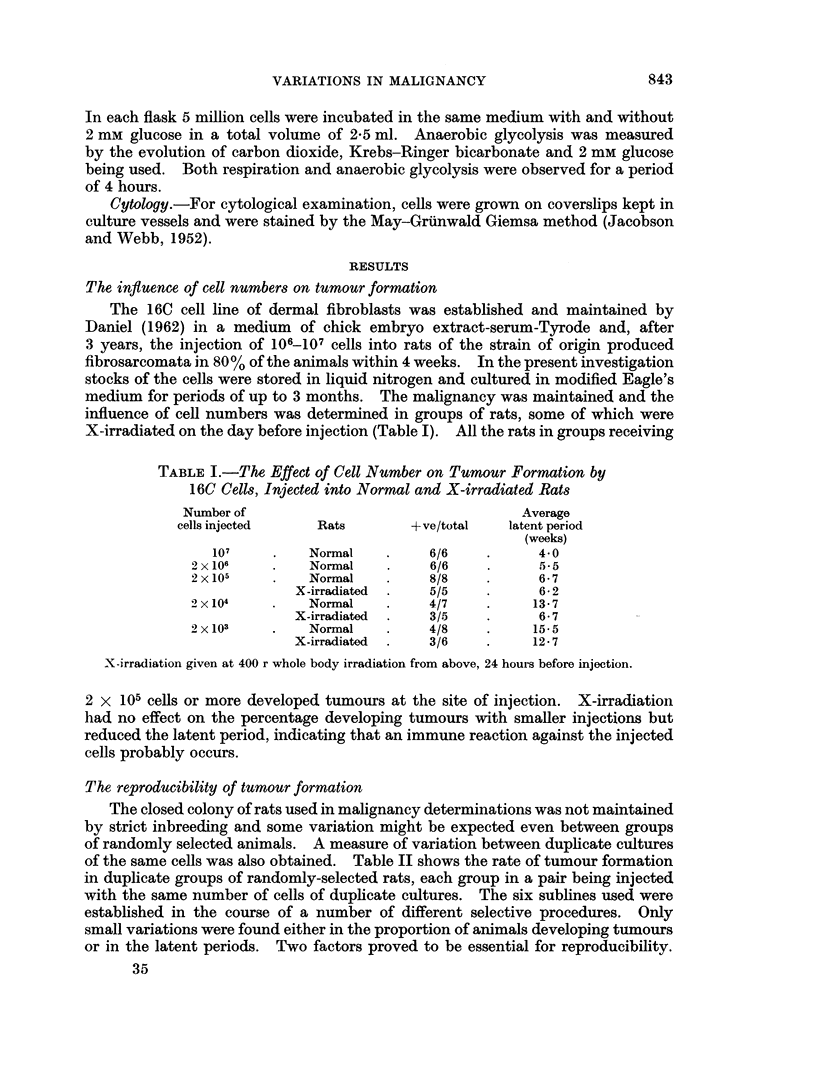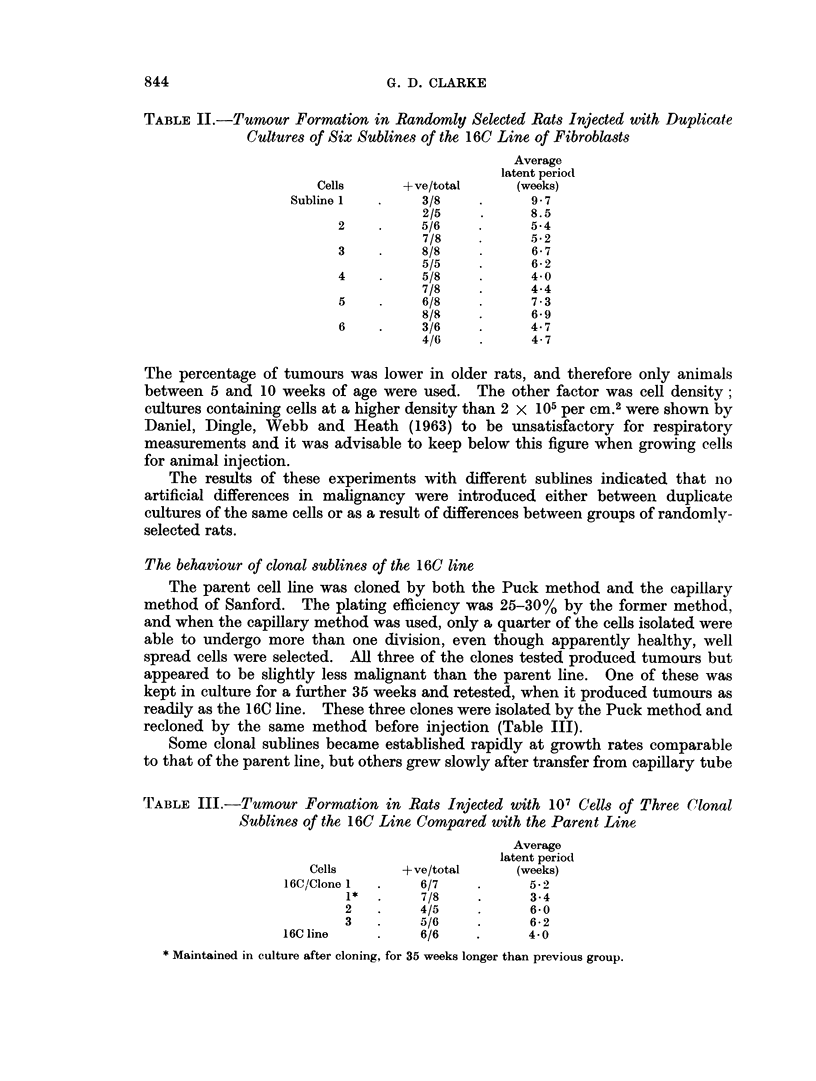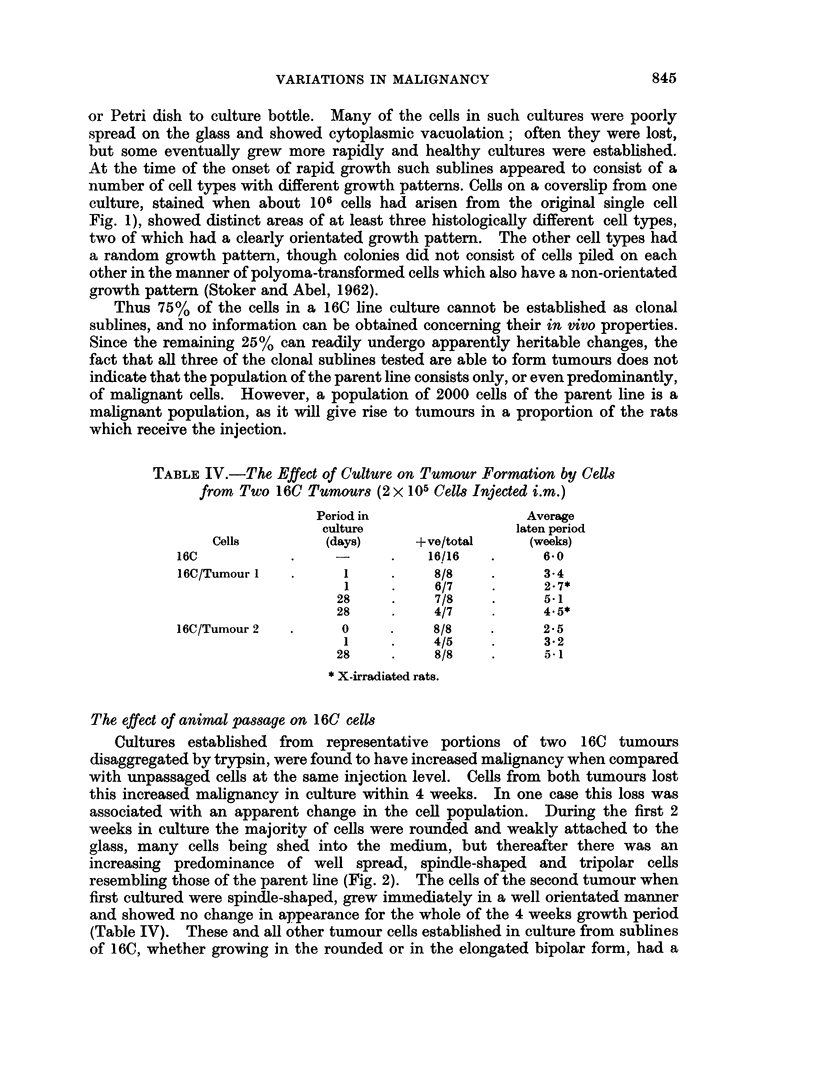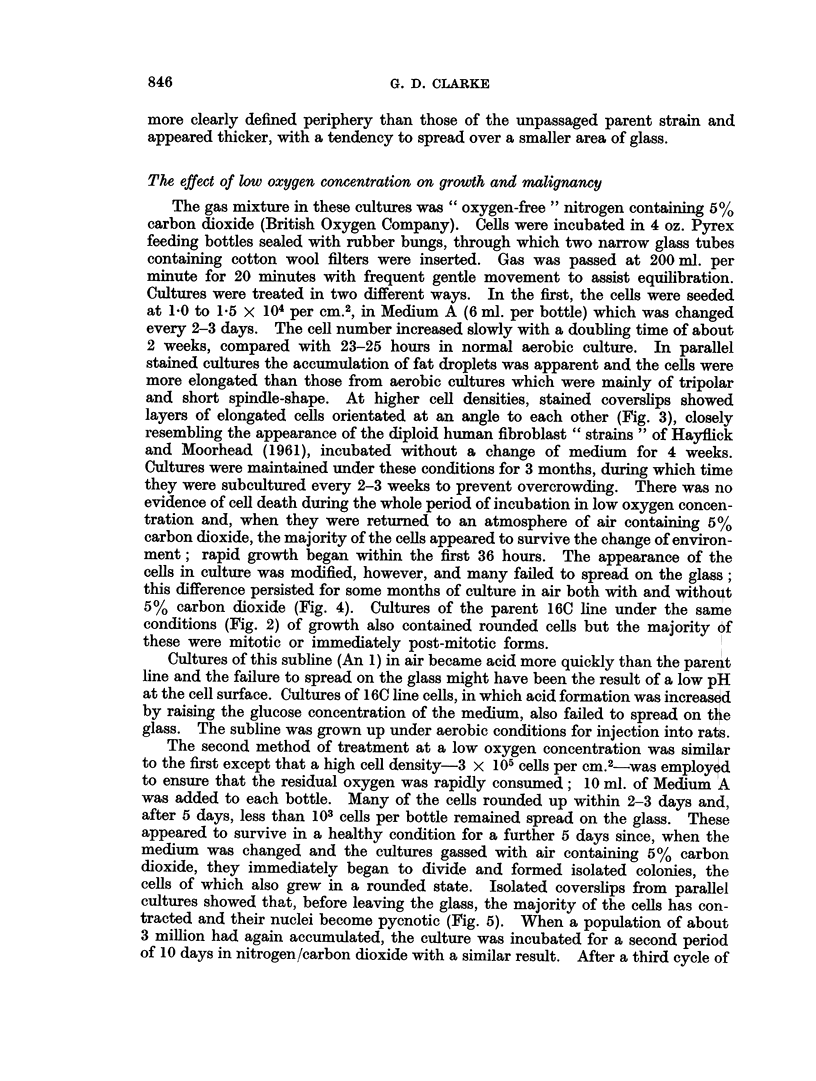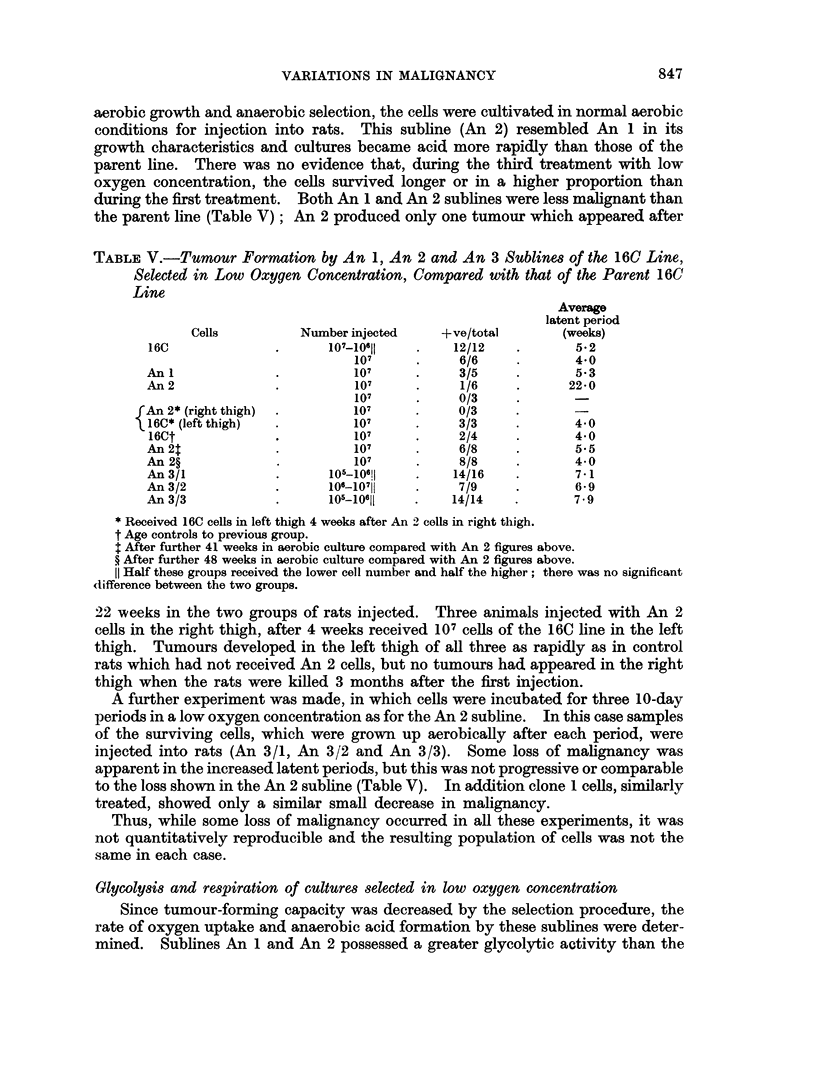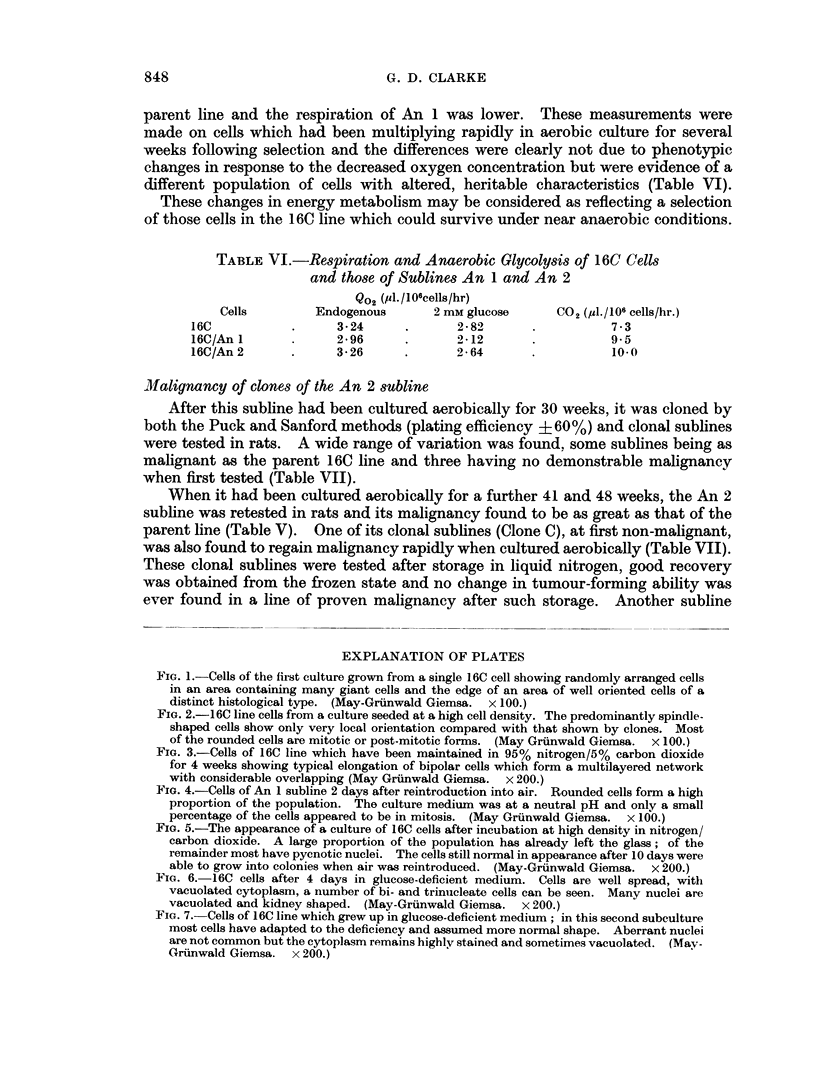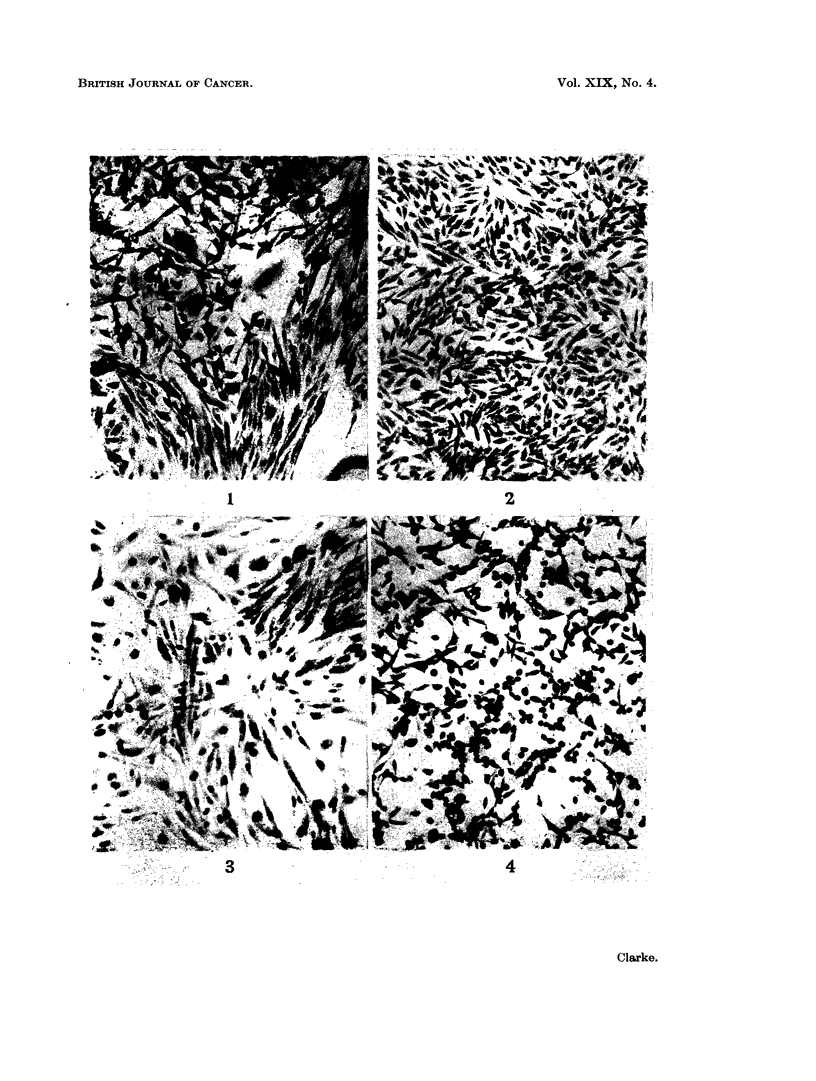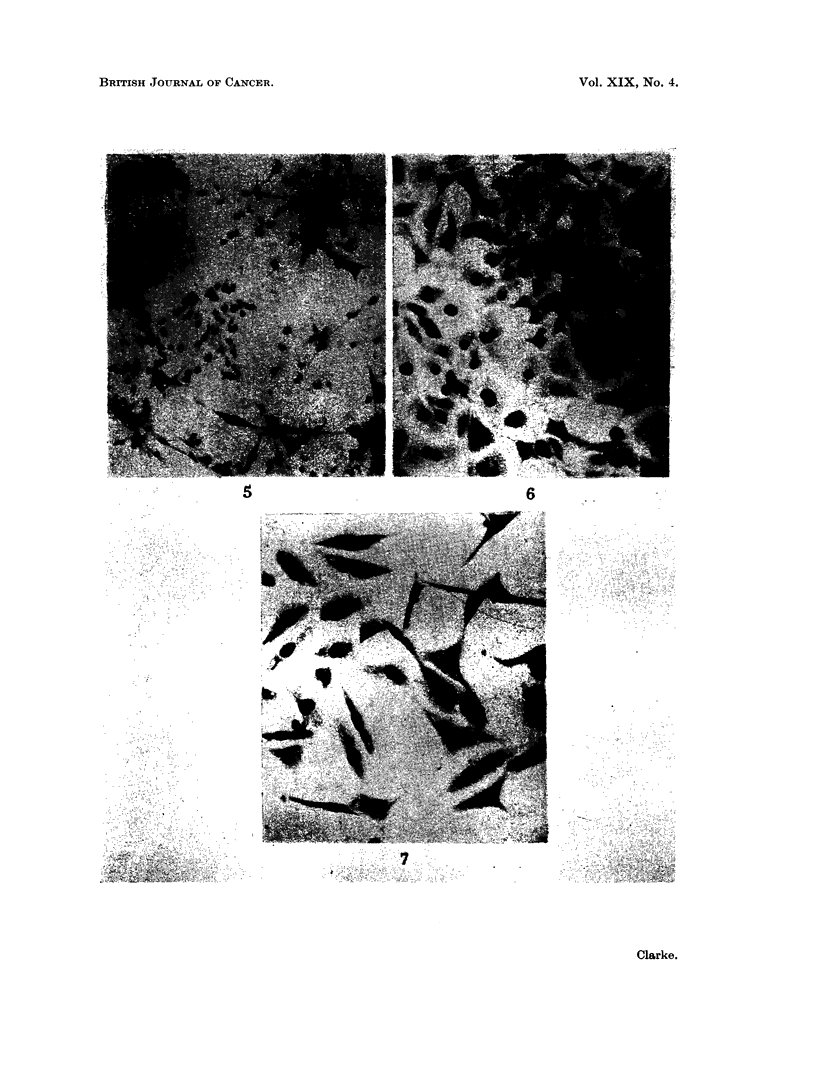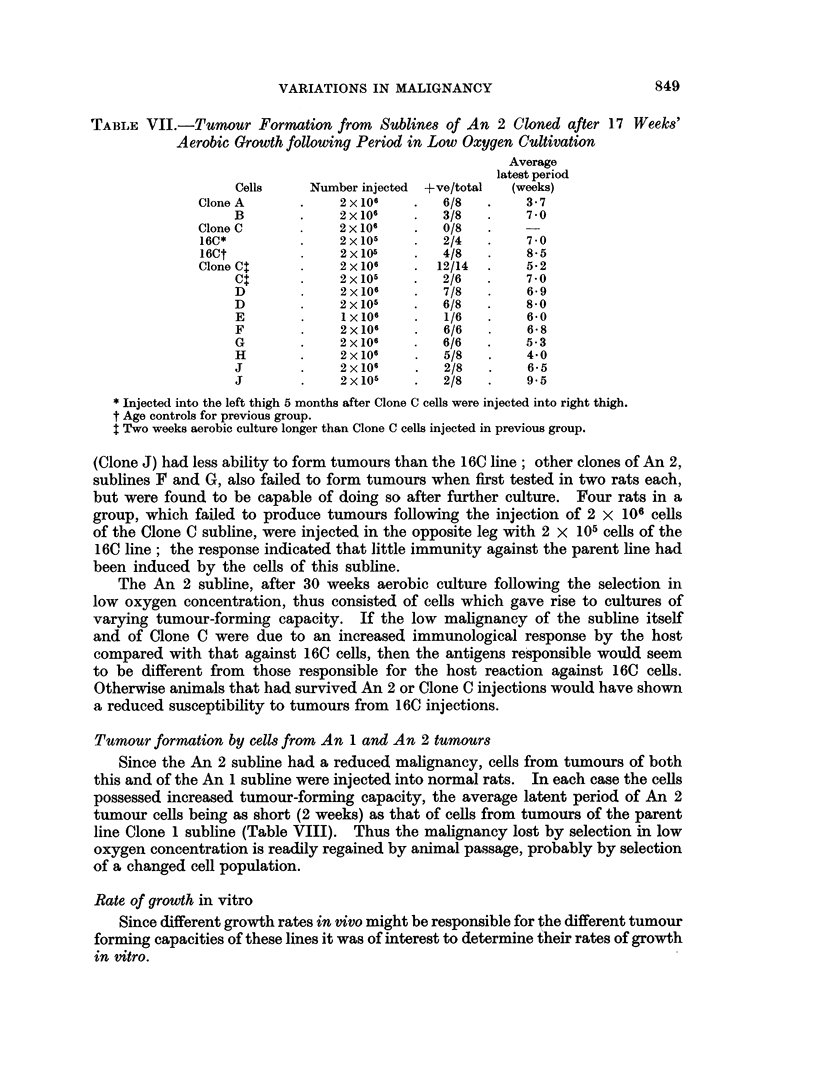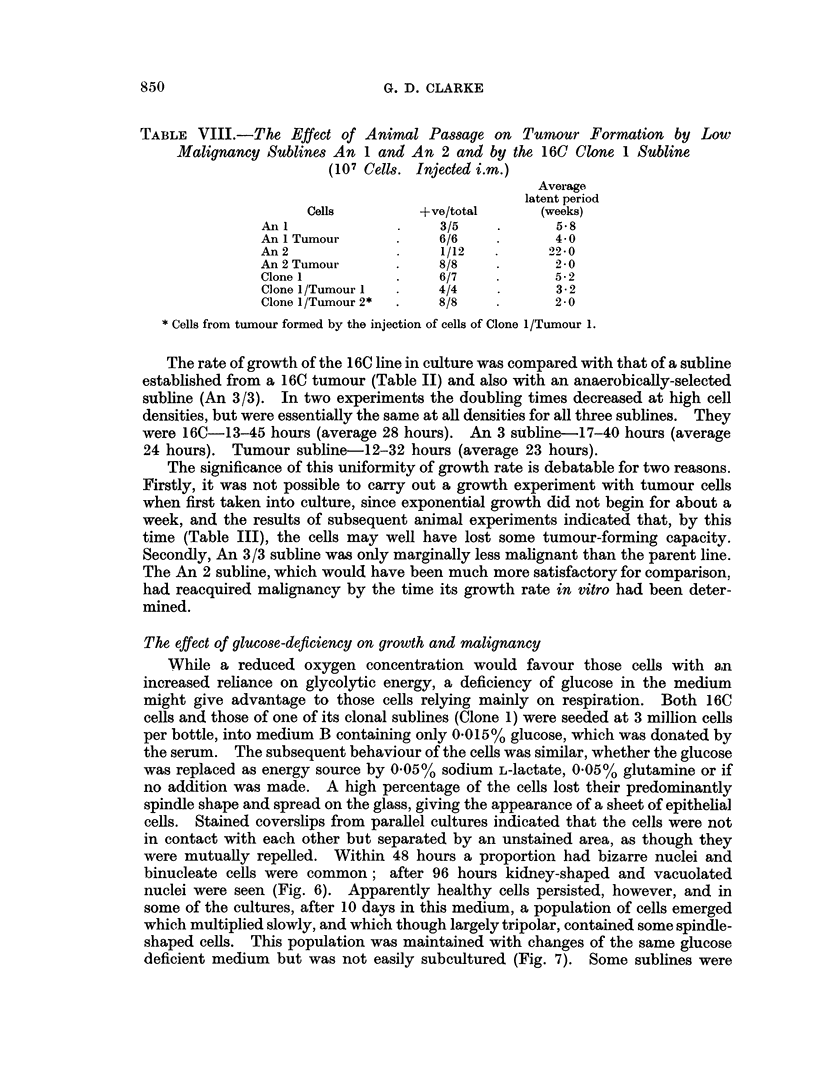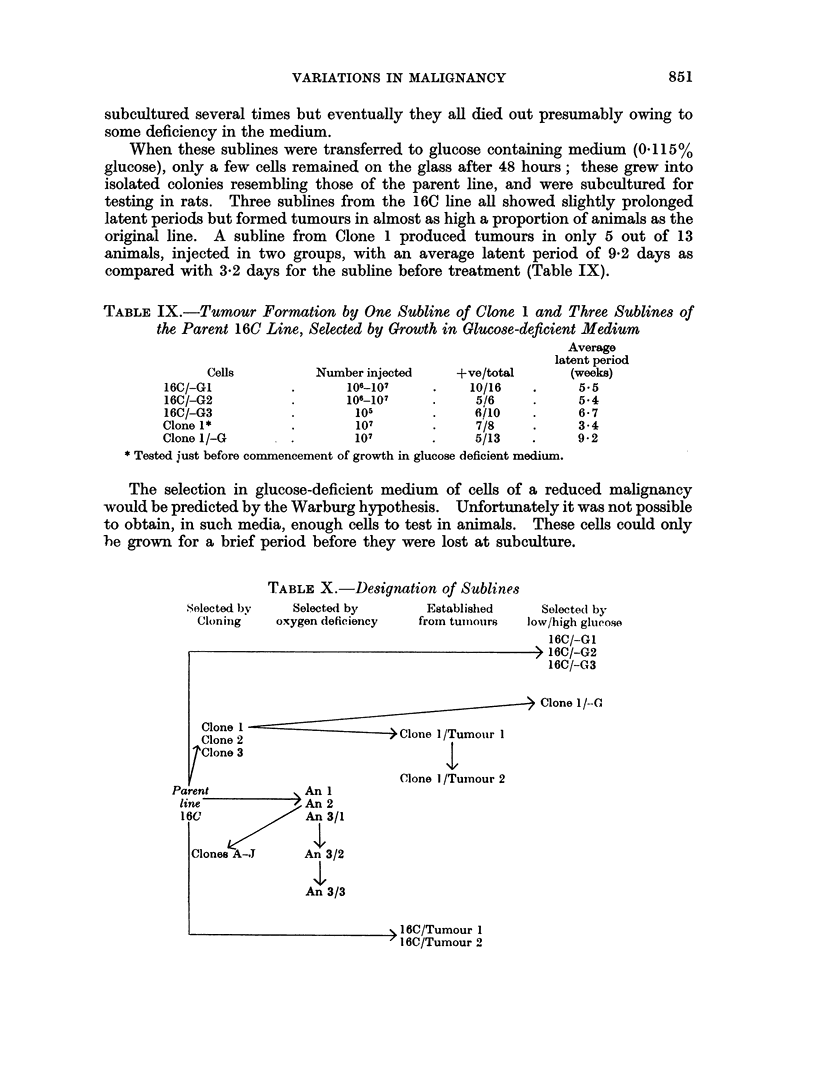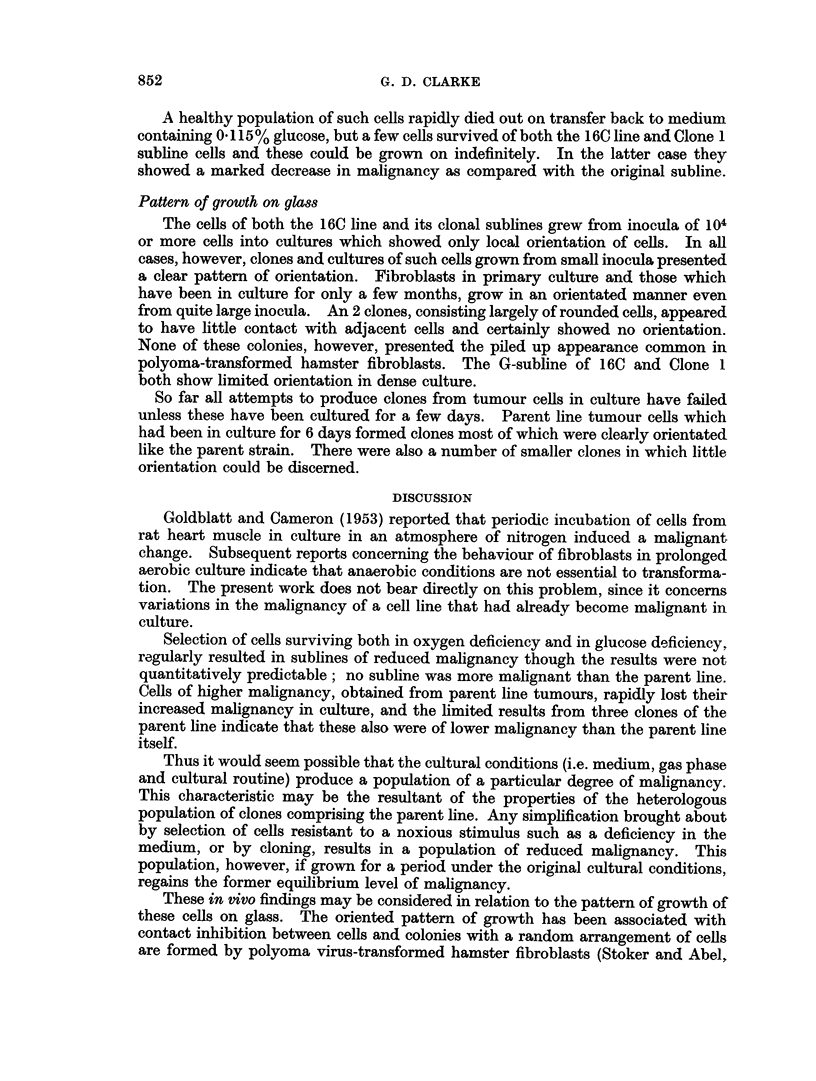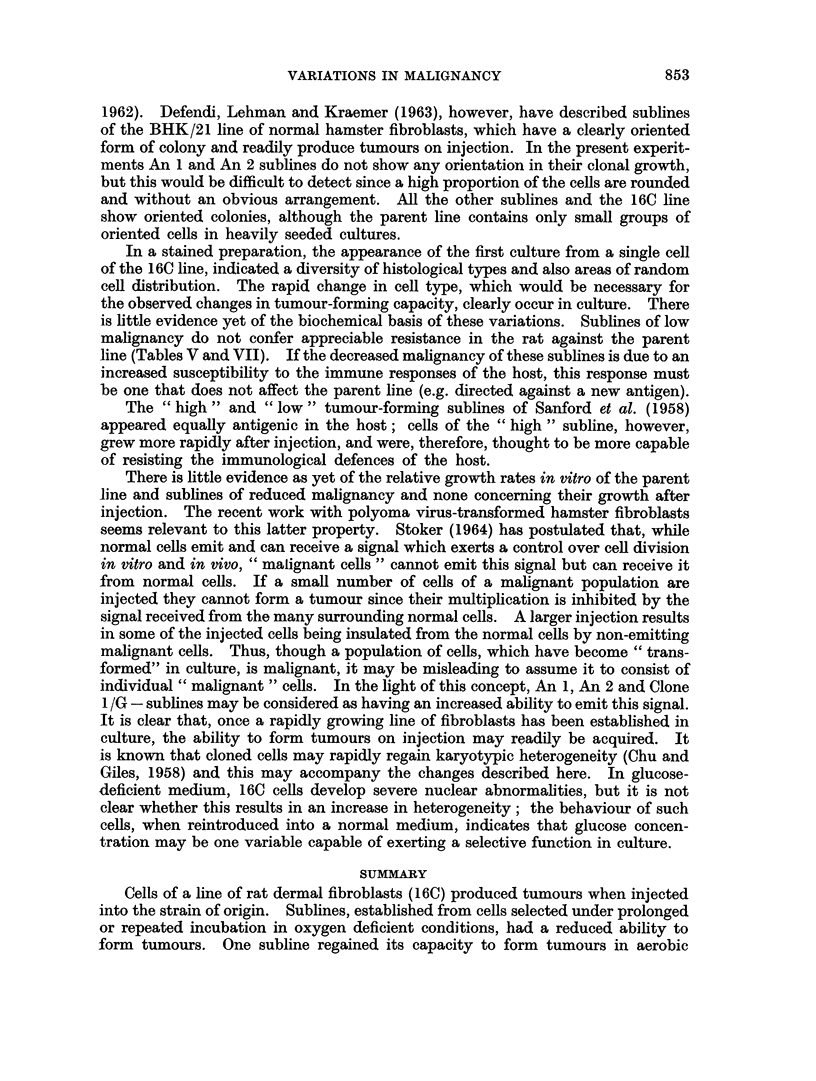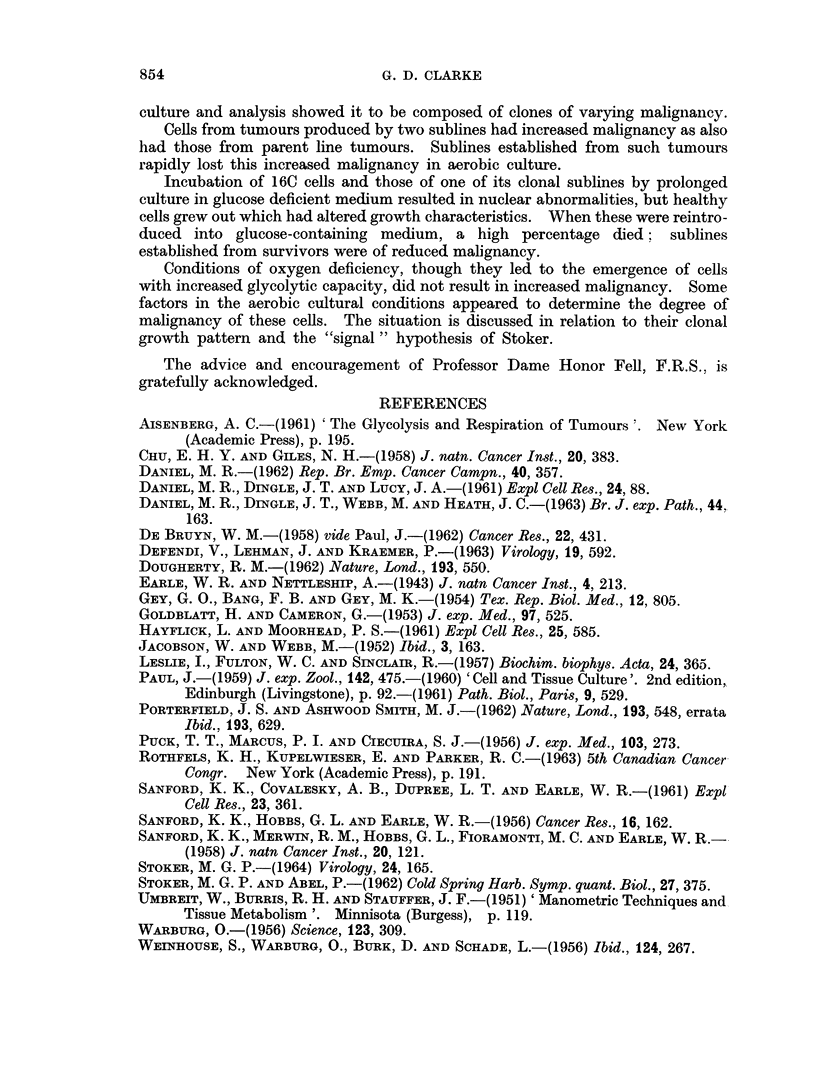# Variations in the tumour-forming capacity of a line of rat fibroblasts (16C) following selection in vitro.

**DOI:** 10.1038/bjc.1965.97

**Published:** 1965-12

**Authors:** G. D. Clarke

## Abstract

**Images:**


### VARIATIONS IN THE TUMOUR-FORMING CAPACITY OF A LINE OF RAT FIBROBLASTS (16C) FOLLOWING SELECTION *IN VITRO*

G. D. CLARKE*

*From the Strangeways Research Laboratory, Cambridge*

Received for publication July 10, 1965

THE Warburg hypothesis of malignant change proposed that an irreversible respiratory lesion in a cell is followed by a compensating increase in the ability to derive energy by the glycolytic breakdown of glucose. This process is assumed to be unaffected by the mechanism of respiratory inhibition, which Warburg visualises as an essential part of the normal control of cell division (Warburg, 1956; Weinhouse, Warburg, Burk and Schade, 1956). The many *in vitro* biochemical observations, comparing aspects of the respiration and glycolysis of normal and tumour tissue, are difficult to evaluate (Aisenberg, 1961). Comparison cannot be made *in vitro* under the exact conditions prevailing in the tissues and there is evidence that some environmental factors are critical. Paul (1959) showed that the hydrogen ion concentration of the environment in which cells had been grown affects the relative rates of respiration and glycolysis. Moreover (Paul, 1961), the effect of oxygen concentration is such that observed differences in the rates of uptake of oxygen and lactic acid formation by slices of normal and tumour tissue *in vitro* could be due to a restricted supply of oxygen in tumours. Leslie, Fulton and Sinclair (1957) in experiments designed to study differences in glucose metabolism, showed that the short-term behaviour of cells *in vitro* is dictated by a reaction to the wide differences between the new environment and the intracellular concentrations of substrates.

Goldblatt and Cameron (1953) provided a new approach to the problem in demonstrating that rat heart muscle cells, subjected in culture to periods of incubation in nitrogen, gave rise to tumours when injected, together with embryonic heart cells, into the anterior chamber of the eye; control cultures incubated throughout in air produced no tumours. This finding appeared to support the Warburg theory, but many reports now confirm that such a transformation *in vitro* may occur in both mouse and rat fibroblasts in normal aerobic culture without the demonstrable intervention of any carcinogenic agent (Gey, Bang and Gey, 1954; Earle and Nettleship, 1943; Daniel, 1962; Rothfels, Kupelwieser and Parker, 1963). Cells from inbred mice may develop such a malignant potential after six months (Daniel, 1962) and this property may be lost again on continued culture (De Bruyn, 1958; Sanford, Hobbs and Earle, 1956).

If malignant cells derive an essential part of their energy from the glycolytic breakdown of glucose, then it might be expected that conditions which inhibited respiration would favour their preferential survival. Similarly, conditions which inhibited glycolysis would promote the emergence of normal cells. The present investigation concerns the effect of changes in cultural conditions on the malignancy

* Member of the external staff, Medical Research Council.

of a line of rat dermal fibroblasts, as measured by the percentage of animals showing tumours and the time of appearance of these tumours, following the injection of a known number of cells. The cells were cultivated in an atmosphere containing very low oxygen concentration and, alternatively, in medium containing low glucose concentration ; survivors of both procedures were grown subsequently in normal aerobic conditions for testing in rats.

### MATERIALS AND METHODS

*Cells.*—The line of fibroblasts used for these experiments was the 16C line established in culture by Dr. Mary Daniel of this laboratory from the dorsal skin of foetal rats near term (Daniel, Dingle and Lucy, 1961). The rats from which the cultures were derived and on which the malignancy of sublines was tested, were of the Strangeways hooded strain which has been maintained as a closed colony for 30 years. The cells had been cultivated in medium of Tyrode's solution, serum (equine or bovine) and chick embryo extract in the ratio 6 : 3 : 1 for 3 years before this investigation began and had already been shown to be capable of forming tumours in rats (Daniel, 1962). A stock of these 16C cells was stored subsequently in liquid nitrogen in medium containing 15% bovine serum and 10% dimethyl sulphoxide (Dougherty, 1962 ; Porterfield and Ashwood-Smith, 1962), and cultures from this stock were maintained for up to 3 months at a time in modified Eagle's medium.

*Culture conditions.*—The following media were used, Medium A : Eagle's medium made up according to Paul (1960) and containing 10% bovine serum and 0·1% glucose. Medium B : the same as A but with 15% serum, 0·1 mg. per ml. each of L-serine and L-aspartic acid and with a three-fold concentration of growth factors (Solution 4). Where not otherwise stated, the gas phase was air ; when 5% carbon dioxide was used the bicarbonate concentration was increased to 2·2 mg. per ml. No antibiotics were used in long term cultures, but 100 u per ml. penicillin and 100 $\mu$g. per ml. of streptomycin were added in the first culture from fresh tissues or from cells stored in liquid nitrogen.

Cells were maintained in 4 oz. Pyrex feeding bottles or medical flats, medium being changed usually every 2–3 days and sub-cultures made when a complete monolayer had been formed—generally every 7–10 days. The medium was removed and the cell sheet treated with a 0·25% solution of trypsin in Tyrode's solution without glucose for 5 minutes, the cell suspension was mixed with an equal volume of medium, centrifuged at 1000 r.p.m., suspended in medium and re-inoculated at about $10^4$ cells per ml. into 4 oz. feeding bottles or medical flats.

*Cloning procedures.*—The usual method employed was based on that of Puck, Marcus and Ciecuira (1956) ; 10 to 500 cells from a single cell suspension were seeded into 60 mm. resistance glass Petri dishes in Medium B and incubated in a desiccator until colonies of sufficient size were formed. It was preferable to use medium with the usual 0·37 mg. per ml. of bicarbonate but to gas the desiccator briefly with 5% carbon dioxide in air. Not only did the cells grow faster at pH 6·9–7·1 but, as the medium did not become alkaline so quickly when the dishes were handled, the risk of cell detachment was reduced. The cells of individual colonies were first isolated with penicillin cups attached to the glass with silicone grease and then removed with 0·25% trypsin. The cells were seeded into culture vessels in Medium B and the medium was changed after 18 hours. In cloning by this method care was taken to ensure that a suspension of single cells was obtained (not a difficult procedure with the 16C line) and that the subsequent dilution and seeding was carried out quickly to avoid reclumping.

The second method, used to obtain clones from one subline, was similar to the capillary method of Sanford *et al.* (1961). Cells at the appropriate dilution were sucked into 50–100 mm. lengths of 0·1 mm. i.d. Pyrex tubing, the ends were sealed in a micro-burner, and the tubes were then incubated in a sterile Petri dish until the cells appeared to be well spread on the glass (6–10 hours). Lengths of 3–5 mm. were cut under the microscope so that each contained a single cell, care being taken to examine the tube from a number of angles to ensure that not more than one cell was present. Tubes were transferred individually to 2 ml. screw-capped culture bottles containing 0·2 ml. Medium B and incubated until cells had migrated well out of one end of the tube. They were then removed with 0·25% trypsin, transferred to a normal culture bottle and maintained until about $10^7$ cells had been produced. Ampoules of each clonal subline were then stored in liquid nitrogen.

*Injection of cultures.*—Cells for animal injection were grown in 4 oz. medical flats or 1 litre Roux bottles, washed rapidly with Tyrode's solution and treated with trypsin in the usual manner. Final dilutions were made with culture medium, such that all injections were given in a volume of 0·5 ml. The cells were injected as soon as possible after being placed in suspension to avoid exhaustion of the suspending medium and excessive formation of acid, which may occur with higher cell densities. Male rats 5–10 weeks of age received 0·5 ml. of the preparation intramuscularly in the inside of the right thigh. They were examined weekly for tumours and the latent period was recorded as the time at which swelling was first noticed at the site of injection. No case of the regression of a tumour was observed. In groups with long latent periods, tumourless rats were kept for 5 months before being discarded ; tumourless groups were kept for a year.

For some experiments X-irradiated rats were used, the animals being exposed to 400 r. whole body irradiation on the day before injection. Radiation factors were 200 kv X-rays at 15 mA, FSD 50 cm., filtration 1·0 mm. Al and 0·5 mm. Cu ; exposure rate 6·0 r./min.

*Cultivation of tumours.*—Cultures were established from tumours of about 1 cm. diameter ; representative pieces of tissue were removed aseptically and digested with 1% solution of trypsin for about 30 minutes with frequent agitation. After being washed with medium, the cells were seeded at $10^4$–$10^5$ per ml. ; a high percentage usually attached to the glass and a rapidly growing culture was soon established. Cells direct from trypsin-treated tumours did not survive well in the frozen state, but samples were always placed in liquid nitrogen within 10 days of first culture.

*Estimation of growth-rate.*—The growth rate was determined in 4 or 8 oz. feeding bottles inoculated with 1 or $2 \times 10^5$ cells, and cells from 2–3 bottles were counted at intervals over a period of 4–5 days, a haemocytometer with an Improved Neubauer ruling being used. Between 600 and 1000 cells were counted for each determination.

*Respiration and glycolysis.*—Respiration was measured in the Warburg apparatus ; cells were removed from culture vessels containing less than $2 \times 10^5$ cells per cm.$^2$, with 0·25% trypsin solution, and washed in Krebs–Ringer phosphate containing 10% neutralised horse serum (Umbreit, Burris and Stauffer, 1951).

In each flask 5 million cells were incubated in the same medium with and without 2 mM glucose in a total volume of 2·5 ml. Anaerobic glycolysis was measured by the evolution of carbon dioxide, Krebs–Ringer bicarbonate and 2 mM glucose being used. Both respiration and anaerobic glycolysis were observed for a period of 4 hours.

*Cytology.*—For cytological examination, cells were grown on coverslips kept in culture vessels and were stained by the May–Grünwald Giemsa method (Jacobson and Webb, 1952).

### RESULTS

### *The influence of cell numbers on tumour formation*

The 16C cell line of dermal fibroblasts was established and maintained by Daniel (1962) in a medium of chick embryo extract-serum-Tyrode and, after 3 years, the injection of $10^6$–$10^7$ cells into rats of the strain of origin produced fibrosarcomata in 80% of the animals within 4 weeks. In the present investigation stocks of the cells were stored in liquid nitrogen and cultured in modified Eagle's medium for periods of up to 3 months. The malignancy was maintained and the influence of cell numbers was determined in groups of rats, some of which were X-irradiated on the day before injection (Table I). All the rats in groups receiving $2 \times 10^5$ cells or more developed tumours at the site of injection. X-irradiation had no effect on the percentage developing tumours with smaller injections but reduced the latent period, indicating that an immune reaction against the injected cells probably occurs.

TABLE I.—*The Effect of Cell Number on Tumour Formation by 16C Cells, Injected into Normal and X-irradiated Rats*

| Number of cells injected | Rats | +ve/total | Average latent period (weeks) |
|---|---|---|---|
| $10^7$ | Normal | 6/6 | 4·0 |
| $2 \times 10^6$ | Normal | 6/6 | 5·5 |
| $2 \times 10^5$ | Normal | 8/8 | 6·7 |
| | X-irradiated | 5/5 | 6·2 |
| $2 \times 10^4$ | Normal | 4/7 | 13·7 |
| | X-irradiated | 3/5 | 6·7 |
| $2 \times 10^3$ | Normal | 4/8 | 15·5 |
| | X-irradiated | 3/6 | 12·7 |

X-irradiation given at 400 r whole body irradiation from above, 24 hours before injection.

### *The reproducibility of tumour formation*

The closed colony of rats used in malignancy determinations was not maintained by strict inbreeding and some variation might be expected even between groups of randomly selected animals. A measure of variation between duplicate cultures of the same cells was also obtained. Table II shows the rate of tumour formation in duplicate groups of randomly-selected rats, each group in a pair being injected with the same number of cells of duplicate cultures. The six sublines used were established in the course of a number of different selective procedures. Only small variations were found either in the proportion of animals developing tumours or in the latent periods. Two factors proved to be essential for reproducibility.

TABLE II.—*Tumour Formation in Randomly Selected Rats Injected with Duplicate Cultures of Six Sublines of the* 16C *Line of Fibroblasts*

| Cells | +ve/total | Average latent period (weeks) |
|---|---|---|
| Subline 1 | 3/8 | 9·7 |
| | 2/5 | 8.5 |
| 2 | 5/6 | 5·4 |
| | 7/8 | 5·2 |
| 3 | 8/8 | 6·7 |
| | 5/5 | 6·2 |
| 4 | 5/8 | 4·0 |
| | 7/8 | 4·4 |
| 5 | 6/8 | 7·3 |
| | 8/8 | 6·9 |
| 6 | 3/6 | 4·7 |
| | 4/6 | 4·7 |

The percentage of tumours was lower in older rats, and therefore only animals between 5 and 10 weeks of age were used. The other factor was cell density; cultures containing cells at a higher density than $2 \times 10^5$ per $cm.^2$ were shown by Daniel, Dingle, Webb and Heath (1963) to be unsatisfactory for respiratory measurements and it was advisable to keep below this figure when growing cells for animal injection.

The results of these experiments with different sublines indicated that no artificial differences in malignancy were introduced either between duplicate cultures of the same cells or as a result of differences between groups of randomly-selected rats.

*The behaviour of clonal sublines of the* 16C *line*

The parent cell line was cloned by both the Puck method and the capillary method of Sanford. The plating efficiency was 25–30% by the former method, and when the capillary method was used, only a quarter of the cells isolated were able to undergo more than one division, even though apparently healthy, well spread cells were selected. All three of the clones tested produced tumours but appeared to be slightly less malignant than the parent line. One of these was kept in culture for a further 35 weeks and retested, when it produced tumours as readily as the 16C line. These three clones were isolated by the Puck method and recloned by the same method before injection (Table III).

Some clonal sublines became established rapidly at growth rates comparable to that of the parent line, but others grew slowly after transfer from capillary tube

TABLE III.—*Tumour Formation in Rats Injected with* $10^7$ *Cells of Three Clonal Sublines of the* 16C *Line Compared with the Parent Line*

| Cells | +ve/total | Average latent period (weeks) |
|---|---|---|
| 16C/Clone 1 | 6/7 | 5·2 |
| 1* | 7/8 | 3·4 |
| 2 | 4/5 | 6·0 |
| 3 | 5/6 | 6·2 |
| 16C line | 6/6 | 4·0 |

* Maintained in culture after cloning, for 35 weeks longer than previous group.

or Petri dish to culture bottle. Many of the cells in such cultures were poorly spread on the glass and showed cytoplasmic vacuolation ; often they were lost, but some eventually grew more rapidly and healthy cultures were established. At the time of the onset of rapid growth such sublines appeared to consist of a number of cell types with different growth patterns. Cells on a coverslip from one culture, stained when about $10^6$ cells had arisen from the original single cell Fig. 1), showed distinct areas of at least three histologically different cell types, two of which had a clearly orientated growth pattern. The other cell types had a random growth pattern, though colonies did not consist of cells piled on each other in the manner of polyoma-transformed cells which also have a non-orientated growth pattern (Stoker and Abel, 1962).

Thus 75% of the cells in a 16C line culture cannot be established as clonal sublines, and no information can be obtained concerning their *in vivo* properties. Since the remaining 25% can readily undergo apparently heritable changes, the fact that all three of the clonal sublines tested are able to form tumours does not indicate that the population of the parent line consists only, or even predominantly, of malignant cells. However, a population of 2000 cells of the parent line is a malignant population, as it will give rise to tumours in a proportion of the rats which receive the injection.

TABLE IV.—*The Effect of Culture on Tumour Formation by Cells from Two 16C Tumours* ($2 \times 10^5$ *Cells Injected i.m.*)

| Cells | Period in culture (days) | +ve/total | Average laten period (weeks) |
|---|---|---|---|
| 16C | — | 16/16 | 6·0 |
| 16C/Tumour 1 | 1 | 8/8 | 3·4 |
| | 1 | 6/7 | 2·7* |
| | 28 | 7/8 | 5·1 |
| | 28 | 4/7 | 4·5* |
| 16C/Tumour 2 | 0 | 8/8 | 2·5 |
| | 1 | 4/5 | 3·2 |
| | 28 | 8/8 | 5·1 |

* X-irradiated rats.

*The effect of animal passage on 16C cells*

Cultures established from representative portions of two 16C tumours disaggregated by trypsin, were found to have increased malignancy when compared with unpassaged cells at the same injection level. Cells from both tumours lost this increased malignancy in culture within 4 weeks. In one case this loss was associated with an apparent change in the cell population. During the first 2 weeks in culture the majority of cells were rounded and weakly attached to the glass, many cells being shed into the medium, but thereafter there was an increasing predominance of well spread, spindle-shaped and tripolar cells resembling those of the parent line (Fig. 2). The cells of the second tumour when first cultured were spindle-shaped, grew immediately in a well orientated manner and showed no change in appearance for the whole of the 4 weeks growth period (Table IV). These and all other tumour cells established in culture from sublines of 16C, whether growing in the rounded or in the elongated bipolar form, had a more clearly defined periphery than those of the unpassaged parent strain and appeared thicker, with a tendency to spread over a smaller area of glass.

*The effect of low oxygen concentration on growth and malignancy*

The gas mixture in these cultures was " oxygen-free " nitrogen containing 5% carbon dioxide (British Oxygen Company). Cells were incubated in 4 oz. Pyrex feeding bottles sealed with rubber bungs, through which two narrow glass tubes containing cotton wool filters were inserted. Gas was passed at 200 ml. per minute for 20 minutes with frequent gentle movement to assist equilibration. Cultures were treated in two different ways. In the first, the cells were seeded at 1·0 to 1·5 $\times$ $10^4$ per cm.$^2$, in Medium A (6 ml. per bottle) which was changed every 2–3 days. The cell number increased slowly with a doubling time of about 2 weeks, compared with 23–25 hours in normal aerobic culture. In parallel stained cultures the accumulation of fat droplets was apparent and the cells were more elongated than those from aerobic cultures which were mainly of tripolar and short spindle-shape. At higher cell densities, stained coverslips showed layers of elongated cells orientated at an angle to each other (Fig. 3), closely resembling the appearance of the diploid human fibroblast " strains " of Hayflick and Moorhead (1961), incubated without a change of medium for 4 weeks. Cultures were maintained under these conditions for 3 months, during which time they were subcultured every 2–3 weeks to prevent overcrowding. There was no evidence of cell death during the whole period of incubation in low oxygen concentration and, when they were returned to an atmosphere of air containing 5% carbon dioxide, the majority of the cells appeared to survive the change of environment; rapid growth began within the first 36 hours. The appearance of the cells in culture was modified, however, and many failed to spread on the glass; this difference persisted for some months of culture in air both with and without 5% carbon dioxide (Fig. 4). Cultures of the parent 16C line under the same conditions (Fig. 2) of growth also contained rounded cells but the majority of these were mitotic or immediately post-mitotic forms.

Cultures of this subline (An 1) in air became acid more quickly than the parent line and the failure to spread on the glass might have been the result of a low pH at the cell surface. Cultures of 16C line cells, in which acid formation was increased by raising the glucose concentration of the medium, also failed to spread on the glass. The subline was grown up under aerobic conditions for injection into rats.

The second method of treatment at a low oxygen concentration was similar to the first except that a high cell density—3 $\times$ $10^5$ cells per cm.$^2$—was employed to ensure that the residual oxygen was rapidly consumed; 10 ml. of Medium A was added to each bottle. Many of the cells rounded up within 2–3 days and, after 5 days, less than $10^3$ cells per bottle remained spread on the glass. These appeared to survive in a healthy condition for a further 5 days since, when the medium was changed and the cultures gassed with air containing 5% carbon dioxide, they immediately began to divide and formed isolated colonies, the cells of which also grew in a rounded state. Isolated coverslips from parallel cultures showed that, before leaving the glass, the majority of the cells has contracted and their nuclei become pycnotic (Fig. 5). When a population of about 3 million had again accumulated, the culture was incubated for a second period of 10 days in nitrogen/carbon dioxide with a similar result. After a third cycle of aerobic growth and anaerobic selection, the cells were cultivated in normal aerobic conditions for injection into rats. This subline (An 2) resembled An 1 in its growth characteristics and cultures became acid more rapidly than those of the parent line. There was no evidence that, during the third treatment with low oxygen concentration, the cells survived longer or in a higher proportion than during the first treatment. Both An 1 and An 2 sublines were less malignant than the parent line (Table V); An 2 produced only one tumour which appeared after

TABLE V.—*Tumour Formation by An 1, An 2 and An 3 Sublines of the 16C Line, Selected in Low Oxygen Concentration, Compared with that of the Parent 16C Line*

| Cells | Number injected | +ve/total | Average latent period (weeks) |
|---|---|---|---|
| 16C | $10^7$–$10^6$‖ | 12/12 | 5·2 |
| | $10^7$ | 6/6 | 4·0 |
| An 1 | $10^7$ | 3/5 | 5·3 |
| An 2 | $10^7$ | 1/6 | 22·0 |
| | $10^7$ | 0/3 | — |
| { An 2* (right thigh) | $10^7$ | 0/3 | — |
| { 16C* (left thigh) | $10^7$ | 3/3 | 4·0 |
| 16C† | $10^7$ | 2/4 | 4·0 |
| An 2‡ | $10^7$ | 6/8 | 5·5 |
| An 2§ | $10^7$ | 8/8 | 4·0 |
| An 3/1 | $10^5$–$10^6$‖ | 14/16 | 7·1 |
| An 3/2 | $10^6$–$10^7$‖ | 7/9 | 6·9 |
| An 3/3 | $10^5$–$10^6$‖ | 14/14 | 7·9 |

* Received 16C cells in left thigh 4 weeks after An 2 cells in right thigh.
† Age controls to previous group.
‡ After further 41 weeks in aerobic culture compared with An 2 figures above.
§ After further 48 weeks in aerobic culture compared with An 2 figures above.
‖ Half these groups received the lower cell number and half the higher; there was no significant difference between the two groups.

22 weeks in the two groups of rats injected. Three animals injected with An 2 cells in the right thigh, after 4 weeks received $10^7$ cells of the 16C line in the left thigh. Tumours developed in the left thigh of all three as rapidly as in control rats which had not received An 2 cells, but no tumours had appeared in the right thigh when the rats were killed 3 months after the first injection.

A further experiment was made, in which cells were incubated for three 10-day periods in a low oxygen concentration as for the An 2 subline. In this case samples of the surviving cells, which were grown up aerobically after each period, were injected into rats (An 3/1, An 3/2 and An 3/3). Some loss of malignancy was apparent in the increased latent periods, but this was not progressive or comparable to the loss shown in the An 2 subline (Table V). In addition clone 1 cells, similarly treated, showed only a similar small decrease in malignancy.

Thus, while some loss of malignancy occurred in all these experiments, it was not quantitatively reproducible and the resulting population of cells was not the same in each case.

*Glycolysis and respiration of cultures selected in low oxygen concentration*

Since tumour-forming capacity was decreased by the selection procedure, the rate of oxygen uptake and anaerobic acid formation by these sublines were determined. Sublines An 1 and An 2 possessed a greater glycolytic activity than the parent line and the respiration of An 1 was lower. These measurements were made on cells which had been multiplying rapidly in aerobic culture for several weeks following selection and the differences were clearly not due to phenotypic changes in response to the decreased oxygen concentration but were evidence of a different population of cells with altered, heritable characteristics (Table VI).

These changes in energy metabolism may be considered as reflecting a selection of those cells in the 16C line which could survive under near anaerobic conditions.

TABLE VI.—*Respiration and Anaerobic Glycolysis of* 16C *Cells and those of Sublines An* 1 *and An* 2

| Cells | $Q_{O_2}$ ($\mu$l./$10^6$cells/hr) Endogenous | 2 mM glucose | $CO_2$ ($\mu$l./$10^6$ cells/hr.) |
|---|---|---|---|
| 16C | 3·24 | 2·82 | 7·3 |
| 16C/An 1 | 2·96 | 2·12 | 9·5 |
| 16C/An 2 | 3·26 | 2·64 | 10·0 |

### *Malignancy of clones of the An 2 subline*

After this subline had been cultured aerobically for 30 weeks, it was cloned by both the Puck and Sanford methods (plating efficiency $\pm 60\%$) and clonal sublines were tested in rats. A wide range of variation was found, some sublines being as malignant as the parent 16C line and three having no demonstrable malignancy when first tested (Table VII).

When it had been cultured aerobically for a further 41 and 48 weeks, the An 2 subline was retested in rats and its malignancy found to be as great as that of the parent line (Table V). One of its clonal sublines (Clone C), at first non-malignant, was also found to regain malignancy rapidly when cultured aerobically (Table VII). These clonal sublines were tested after storage in liquid nitrogen, good recovery was obtained from the frozen state and no change in tumour-forming ability was ever found in a line of proven malignancy after such storage. Another subline

---

EXPLANATION OF PLATES

FIG. 1.—Cells of the first culture grown from a single 16C cell showing randomly arranged cells in an area containing many giant cells and the edge of an area of well oriented cells of a distinct histological type. (May-Grünwald Giemsa. $\times 100$.)

FIG. 2.—16C line cells from a culture seeded at a high cell density. The predominantly spindle-shaped cells show only very local orientation compared with that shown by clones. Most of the rounded cells are mitotic or post-mitotic forms. (May Grünwald Giemsa. $\times 100$.)

FIG. 3.—Cells of 16C line which have been maintained in 95% nitrogen/5% carbon dioxide for 4 weeks showing typical elongation of bipolar cells which form a multilayered network with considerable overlapping (May Grünwald Giemsa. $\times 200$.)

FIG. 4.—Cells of An 1 subline 2 days after reintroduction into air. Rounded cells form a high proportion of the population. The culture medium was at a neutral pH and only a small percentage of the cells appeared to be in mitosis. (May Grünwald Giemsa. $\times 100$.)

FIG. 5.—The appearance of a culture of 16C cells after incubation at high density in nitrogen/carbon dioxide. A large proportion of the population has already left the glass; of the remainder most have pycnotic nuclei. The cells still normal in appearance after 10 days were able to grow into colonies when air was reintroduced. (May-Grünwald Giemsa. $\times 200$.)

FIG. 6.—16C cells after 4 days in glucose-deficient medium. Cells are well spread, with vacuolated cytoplasm, a number of bi- and trinucleate cells can be seen. Many nuclei are vacuolated and kidney shaped. (May-Grünwald Giemsa. $\times 200$.)

FIG. 7.—Cells of 16C line which grew up in glucose-deficient medium; in this second subculture most cells have adapted to the deficiency and assumed more normal shape. Aberrant nuclei are not common but the cytoplasm remains highly stained and sometimes vacuolated. (May-Grünwald Giemsa. $\times 200$.)

1 2 3 4

Clarke.

5

6

7

TABLE VII.—*Tumour Formation from Sublines of An* 2 *Cloned after* 17 *Weeks' Aerobic Growth following Period in Low Oxygen Cultivation*

| Cells | Number injected | +ve/total | Average latest period (weeks) |
|---|---|---|---|
| Clone A | $2\times10^6$ | 6/8 | 3·7 |
| B | $2\times10^6$ | 3/8 | 7·0 |
| Clone C | $2\times10^6$ | 0/8 | — |
| 16C* | $2\times10^5$ | 2/4 | 7·0 |
| 16C† | $2\times10^5$ | 4/8 | 8·5 |
| Clone C‡ | $2\times10^6$ | 12/14 | 5·2 |
| C‡ | $2\times10^5$ | 2/6 | 7·0 |
| D | $2\times10^6$ | 7/8 | 6·9 |
| D | $2\times10^5$ | 6/8 | 8·0 |
| E | $1\times10^6$ | 1/6 | 6·0 |
| F | $2\times10^6$ | 6/6 | 6·8 |
| G | $2\times10^6$ | 6/6 | 5·3 |
| H | $2\times10^6$ | 5/8 | 4·0 |
| J | $2\times10^6$ | 2/8 | 6·5 |
| J | $2\times10^5$ | 2/8 | 9·5 |

* Injected into the left thigh 5 months after Clone C cells were injected into right thigh.
† Age controls for previous group.
‡ Two weeks aerobic culture longer than Clone C cells injected in previous group.

(Clone J) had less ability to form tumours than the 16C line ; other clones of An 2, sublines F and G, also failed to form tumours when first tested in two rats each, but were found to be capable of doing so after further culture. Four rats in a group, which failed to produce tumours following the injection of $2 \times 10^6$ cells of the Clone C subline, were injected in the opposite leg with $2 \times 10^5$ cells of the 16C line ; the response indicated that little immunity against the parent line had been induced by the cells of this subline.

The An 2 subline, after 30 weeks aerobic culture following the selection in low oxygen concentration, thus consisted of cells which gave rise to cultures of varying tumour-forming capacity. If the low malignancy of the subline itself and of Clone C were due to an increased immunological response by the host compared with that against 16C cells, then the antigens responsible would seem to be different from those responsible for the host reaction against 16C cells. Otherwise animals that had survived An 2 or Clone C injections would have shown a reduced susceptibility to tumours from 16C injections.

*Tumour formation by cells from An* 1 *and An* 2 *tumours*

Since the An 2 subline had a reduced malignancy, cells from tumours of both this and of the An 1 subline were injected into normal rats. In each case the cells possessed increased tumour-forming capacity, the average latent period of An 2 tumour cells being as short (2 weeks) as that of cells from tumours of the parent line Clone 1 subline (Table VIII). Thus the malignancy lost by selection in low oxygen concentration is readily regained by animal passage, probably by selection of a changed cell population.

*Rate of growth* in vitro

Since different growth rates *in vivo* might be responsible for the different tumour forming capacities of these lines it was of interest to determine their rates of growth *in vitro*.

TABLE VIII.—*The Effect of Animal Passage on Tumour Formation by Low Malignancy Sublines An 1 and An 2 and by the 16C Clone 1 Subline ($10^7$ Cells. Injected i.m.)*

| Cells | +ve/total | Average latent period (weeks) |
|---|---|---|
| An 1 | 3/5 | 5·8 |
| An 1 Tumour | 6/6 | 4·0 |
| An 2 | 1/12 | 22·0 |
| An 2 Tumour | 8/8 | 2·0 |
| Clone 1 | 6/7 | 5·2 |
| Clone 1/Tumour 1 | 4/4 | 3·2 |
| Clone 1/Tumour 2* | 8/8 | 2·0 |

* Cells from tumour formed by the injection of cells of Clone 1/Tumour 1.

The rate of growth of the 16C line in culture was compared with that of a subline established from a 16C tumour (Table II) and also with an anaerobically-selected subline (An 3/3). In two experiments the doubling times decreased at high cell densities, but were essentially the same at all densities for all three sublines. They were 16C—13–45 hours (average 28 hours). An 3 subline—17–40 hours (average 24 hours). Tumour subline—12–32 hours (average 23 hours).

The significance of this uniformity of growth rate is debatable for two reasons. Firstly, it was not possible to carry out a growth experiment with tumour cells when first taken into culture, since exponential growth did not begin for about a week, and the results of subsequent animal experiments indicated that, by this time (Table III), the cells may well have lost some tumour-forming capacity. Secondly, An 3/3 subline was only marginally less malignant than the parent line. The An 2 subline, which would have been much more satisfactory for comparison, had reacquired malignancy by the time its growth rate *in vitro* had been determined.

*The effect of glucose-deficiency on growth and malignancy*

While a reduced oxygen concentration would favour those cells with an increased reliance on glycolytic energy, a deficiency of glucose in the medium might give advantage to those cells relying mainly on respiration. Both 16C cells and those of one of its clonal sublines (Clone 1) were seeded at 3 million cells per bottle, into medium B containing only 0·015% glucose, which was donated by the serum. The subsequent behaviour of the cells was similar, whether the glucose was replaced as energy source by 0·05% sodium L-lactate, 0·05% glutamine or if no addition was made. A high percentage of the cells lost their predominantly spindle shape and spread on the glass, giving the appearance of a sheet of epithelial cells. Stained coverslips from parallel cultures indicated that the cells were not in contact with each other but separated by an unstained area, as though they were mutually repelled. Within 48 hours a proportion had bizarre nuclei and binucleate cells were common; after 96 hours kidney-shaped and vacuolated nuclei were seen (Fig. 6). Apparently healthy cells persisted, however, and in some of the cultures, after 10 days in this medium, a population of cells emerged which multiplied slowly, and which though largely tripolar, contained some spindle-shaped cells. This population was maintained with changes of the same glucose deficient medium but was not easily subcultured (Fig. 7). Some sublines were subcultured several times but eventually they all died out presumably owing to some deficiency in the medium.

When these sublines were transferred to glucose containing medium (0·115% glucose), only a few cells remained on the glass after 48 hours ; these grew into isolated colonies resembling those of the parent line, and were subcultured for testing in rats. Three sublines from the 16C line all showed slightly prolonged latent periods but formed tumours in almost as high a proportion of animals as the original line. A subline from Clone 1 produced tumours in only 5 out of 13 animals, injected in two groups, with an average latent period of 9·2 days as compared with 3·2 days for the subline before treatment (Table IX).

TABLE IX.—*Tumour Formation by One Subline of Clone* 1 *and Three Sublines of the Parent* 16*C Line, Selected by Growth in Glucose-deficient Medium*

| Cells | Number injected | +ve/total | Average latent period (weeks) |
|---|---|---|---|
| 16C/-G1 | $10^6$–$10^7$ | 10/16 | 5·5 |
| 16C/-G2 | $10^6$–$10^7$ | 5/6 | 5·4 |
| 16C/-G3 | $10^5$ | 6/10 | 6·7 |
| Clone 1* | $10^7$ | 7/8 | 3·4 |
| Clone 1/-G | $10^7$ | 5/13 | 9·2 |

* Tested just before commencement of growth in glucose deficient medium.

The selection in glucose-deficient medium of cells of a reduced malignancy would be predicted by the Warburg hypothesis. Unfortunately it was not possible to obtain, in such media, enough cells to test in animals. These cells could only be grown for a brief period before they were lost at subculture.

TABLE X.—*Designation of Sublines*

A healthy population of such cells rapidly died out on transfer back to medium containing 0·115% glucose, but a few cells survived of both the 16C line and Clone 1 subline cells and these could be grown on indefinitely. In the latter case they showed a marked decrease in malignancy as compared with the original subline.

*Pattern of growth on glass*

The cells of both the 16C line and its clonal sublines grew from inocula of $10^4$ or more cells into cultures which showed only local orientation of cells. In all cases, however, clones and cultures of such cells grown from small inocula presented a clear pattern of orientation. Fibroblasts in primary culture and those which have been in culture for only a few months, grow in an orientated manner even from quite large inocula. An 2 clones, consisting largely of rounded cells, appeared to have little contact with adjacent cells and certainly showed no orientation. None of these colonies, however, presented the piled up appearance common in polyoma-transformed hamster fibroblasts. The G-subline of 16C and Clone 1 both show limited orientation in dense culture.

So far all attempts to produce clones from tumour cells in culture have failed unless these have been cultured for a few days. Parent line tumour cells which had been in culture for 6 days formed clones most of which were clearly orientated like the parent strain. There were also a number of smaller clones in which little orientation could be discerned.

### DISCUSSION

Goldblatt and Cameron (1953) reported that periodic incubation of cells from rat heart muscle in culture in an atmosphere of nitrogen induced a malignant change. Subsequent reports concerning the behaviour of fibroblasts in prolonged aerobic culture indicate that anaerobic conditions are not essential to transformation. The present work does not bear directly on this problem, since it concerns variations in the malignancy of a cell line that had already become malignant in culture.

Selection of cells surviving both in oxygen deficiency and in glucose deficiency, regularly resulted in sublines of reduced malignancy though the results were not quantitatively predictable ; no subline was more malignant than the parent line. Cells of higher malignancy, obtained from parent line tumours, rapidly lost their increased malignancy in culture, and the limited results from three clones of the parent line indicate that these also were of lower malignancy than the parent line itself.

Thus it would seem possible that the cultural conditions (i.e. medium, gas phase and cultural routine) produce a population of a particular degree of malignancy. This characteristic may be the resultant of the properties of the heterologous population of clones comprising the parent line. Any simplification brought about by selection of cells resistant to a noxious stimulus such as a deficiency in the medium, or by cloning, results in a population of reduced malignancy. This population, however, if grown for a period under the original cultural conditions, regains the former equilibrium level of malignancy.

These *in vivo* findings may be considered in relation to the pattern of growth of these cells on glass. The oriented pattern of growth has been associated with contact inhibition between cells and colonies with a random arrangement of cells are formed by polyoma virus-transformed hamster fibroblasts (Stoker and Abel,

1962). Defendi, Lehman and Kraemer (1963), however, have described sublines of the BHK/21 line of normal hamster fibroblasts, which have a clearly oriented form of colony and readily produce tumours on injection. In the present experitments An 1 and An 2 sublines do not show any orientation in their clonal growth, but this would be difficult to detect since a high proportion of the cells are rounded and without an obvious arrangement. All the other sublines and the 16C line show oriented colonies, although the parent line contains only small groups of oriented cells in heavily seeded cultures.

In a stained preparation, the appearance of the first culture from a single cell of the 16C line, indicated a diversity of histological types and also areas of random cell distribution. The rapid change in cell type, which would be necessary for the observed changes in tumour-forming capacity, clearly occur in culture. There is little evidence yet of the biochemical basis of these variations. Sublines of low malignancy do not confer appreciable resistance in the rat against the parent line (Tables V and VII). If the decreased malignancy of these sublines is due to an increased susceptibility to the immune responses of the host, this response must be one that does not affect the parent line (e.g. directed against a new antigen).

The " high " and " low " tumour-forming sublines of Sanford *et al.* (1958) appeared equally antigenic in the host; cells of the " high " subline, however, grew more rapidly after injection, and were, therefore, thought to be more capable of resisting the immunological defences of the host.

There is little evidence as yet of the relative growth rates *in vitro* of the parent line and sublines of reduced malignancy and none concerning their growth after injection. The recent work with polyoma virus-transformed hamster fibroblasts seems relevant to this latter property. Stoker (1964) has postulated that, while normal cells emit and can receive a signal which exerts a control over cell division *in vitro* and *in vivo*, " malignant cells " cannot emit this signal but can receive it from normal cells. If a small number of cells of a malignant population are injected they cannot form a tumour since their multiplication is inhibited by the signal received from the many surrounding normal cells. A larger injection results in some of the injected cells being insulated from the normal cells by non-emitting malignant cells. Thus, though a population of cells, which have become " transformed" in culture, is malignant, it may be misleading to assume it to consist of individual " malignant " cells. In the light of this concept, An 1, An 2 and Clone 1/G — sublines may be considered as having an increased ability to emit this signal. It is clear that, once a rapidly growing line of fibroblasts has been established in culture, the ability to form tumours on injection may readily be acquired. It is known that cloned cells may rapidly regain karyotypic heterogeneity (Chu and Giles, 1958) and this may accompany the changes described here. In glucose-deficient medium, 16C cells develop severe nuclear abnormalities, but it is not clear whether this results in an increase in heterogeneity; the behaviour of such cells, when reintroduced into a normal medium, indicates that glucose concentration may be one variable capable of exerting a selective function in culture.

### SUMMARY

Cells of a line of rat dermal fibroblasts (16C) produced tumours when injected into the strain of origin. Sublines, established from cells selected under prolonged or repeated incubation in oxygen deficient conditions, had a reduced ability to form tumours. One subline regained its capacity to form tumours in aerobic culture and analysis showed it to be composed of clones of varying malignancy.

Cells from tumours produced by two sublines had increased malignancy as also had those from parent line tumours. Sublines established from such tumours rapidly lost this increased malignancy in aerobic culture.

Incubation of 16C cells and those of one of its clonal sublines by prolonged culture in glucose deficient medium resulted in nuclear abnormalities, but healthy cells grew out which had altered growth characteristics. When these were reintroduced into glucose-containing medium, a high percentage died; sublines established from survivors were of reduced malignancy.

Conditions of oxygen deficiency, though they led to the emergence of cells with increased glycolytic capacity, did not result in increased malignancy. Some factors in the aerobic cultural conditions appeared to determine the degree of malignancy of these cells. The situation is discussed in relation to their clonal growth pattern and the "signal" hypothesis of Stoker.

The advice and encouragement of Professor Dame Honor Fell, F.R.S., is gratefully acknowledged.

### REFERENCES

AISENBERG, A. C.—(1961) 'The Glycolysis and Respiration of Tumours'. New York (Academic Press), p. 195.
CHU, E. H. Y. AND GILES, N. H.—(1958) *J. natn. Cancer Inst.*, **20**, 383.
DANIEL, M. R.—(1962) *Rep. Br. Emp. Cancer Campn.*, **40**, 357.
DANIEL, M. R., DINGLE, J. T. AND LUCY, J. A.—(1961) *Expl Cell Res.*, **24**, 88.
DANIEL, M. R., DINGLE, J. T., WEBB, M. AND HEATH, J. C.—(1963) *Br. J. exp. Path.*, **44**, 163.
DE BRUYN, W. M.—(1958) *vide* Paul, J.—(1962) *Cancer Res.*, **22**, 431.
DEFENDI, V., LEHMAN, J. AND KRAEMER, P.—(1963) *Virology*, **19**, 592.
DOUGHERTY, R. M.—(1962) *Nature, Lond.*, **193**, 550.
EARLE, W. R. AND NETTLESHIP, A.—(1943) *J. natn Cancer Inst.*, **4**, 213.
GEY, G. O., BANG, F. B. AND GEY, M. K.—(1954) *Tex. Rep. Biol. Med.*, **12**, 805.
GOLDBLATT, H. AND CAMERON, G.—(1953) *J. exp. Med.*, **97**, 525.
HAYFLICK, L. AND MOORHEAD, P. S.—(1961) *Expl Cell Res.*, **25**, 585.
JACOBSON, W. AND WEBB, M.—(1952) *Ibid.*, **3**, 163.
LESLIE, I., FULTON, W. C. AND SINCLAIR, R.—(1957) *Biochim. biophys. Acta*, **24**, 365.
PAUL, J.—(1959) *J. exp. Zool.*, **142**, 475.—(1960) 'Cell and Tissue Culture'. 2nd edition, Edinburgh (Livingstone), p. 92.—(1961) *Path. Biol., Paris*, **9**, 529.
PORTERFIELD, J. S. AND ASHWOOD SMITH, M. J.—(1962) *Nature, Lond.*, **193**, 548, errata *Ibid.*, **193**, 629.
PUCK, T. T., MARCUS, P. I. AND CIECUIRA, S. J.—(1956) *J. exp. Med.*, **103**, 273.
ROTHFELS, K. H., KUPELWIESER, E. AND PARKER, R. C.—(1963) *5th Canadian Cancer Congr.* New York (Academic Press), p. 191.
SANFORD, K. K., COVALESKY, A. B., DUPREE, L. T. AND EARLE, W. R.—(1961) *Expl Cell Res.*, **23**, 361.
SANFORD, K. K., HOBBS, G. L. AND EARLE, W. R.—(1956) *Cancer Res.*, **16**, 162.
SANFORD, K. K., MERWIN, R. M., HOBBS, G. L., FIORAMONTI, M. C. AND EARLE, W. R.—(1958) *J. natn Cancer Inst.*, **20**, 121.
STOKER, M. G. P.—(1964) *Virology*, **24**, 165.
STOKER, M. G. P. AND ABEL, P.—(1962) *Cold Spring Harb. Symp. quant. Biol.*, **27**, 375.
UMBREIT, W., BURRIS, R. H. AND STAUFFER, J. F.—(1951) 'Manometric Techniques and Tissue Metabolism'. Minnisota (Burgess), p. 119.
WARBURG, O.—(1956) *Science*, **123**, 309.
WEINHOUSE, S., WARBURG, O., BURK, D. AND SCHADE, L.—(1956) *Ibid.*, **124**, 267.